# LPI-EnEDT: an ensemble framework with extra tree and decision tree classifiers for imbalanced lncRNA-protein interaction data classification

**DOI:** 10.1186/s13040-021-00277-4

**Published:** 2021-12-03

**Authors:** Lihong Peng, Ruya Yuan, Ling Shen, Pengfei Gao, Liqian Zhou

**Affiliations:** 1 School of Computer Science, Hunan University of Technology, No.88, Taishan West Road, Tianyuan District, Zhuzhou, China; 2 College of Life Sciences and Chemistry, Hunan University of Technology, No.88, Taishan West Road, Tianyuan District, Zhuzhou, China

**Keywords:** lncRNA-protein interaction, Ensemble, Class imbalance

## Abstract

**Background:**

Long noncoding RNAs (lncRNAs) have dense linkages with various biological processes. Identifying interacting lncRNA-protein pairs contributes to understand the functions and mechanisms of lncRNAs. Wet experiments are costly and time-consuming. Most computational methods failed to observe the imbalanced characterize of lncRNA-protein interaction (LPI) data. More importantly, they were measured based on a unique dataset, which produced the prediction bias.

**Results:**

In this study, we develop an Ensemble framework (LPI-EnEDT) with Extra tree and Decision Tree classifiers to implement imbalanced LPI data classification. First, five LPI datasets are arranged. Second, lncRNAs and proteins are separately characterized based on Pyfeat and BioTriangle and concatenated as a vector to represent each lncRNA-protein pair. Finally, an ensemble framework with Extra tree and decision tree classifiers is developed to classify unlabeled lncRNA-protein pairs. The comparative experiments demonstrate that LPI-EnEDT outperforms four classical LPI prediction methods (LPI-BLS, LPI-CatBoost, LPI-SKF, and PLIPCOM) under cross validations on lncRNAs, proteins, and LPIs. The average AUC values on the five datasets are 0.8480, 0,7078, and 0.9066 under the three cross validations, respectively. The average AUPRs are 0.8175, 0.7265, and 0.8882, respectively. Case analyses suggest that there are underlying associations between HOTTIP and Q9Y6M1, NRON and Q15717.

**Conclusions:**

Fusing diverse biological features of lncRNAs and proteins and exploiting an ensemble learning model with Extra tree and decision tree classifiers, this work focus on imbalanced LPI data classification as well as interaction information inference for a new lncRNA (or protein).

**Supplementary Information:**

The online version contains supplementary material available at (10.1186/s13040-021-00277-4).

## Introduction

### Motivation

Noncoding RNAs are molecules regulating various fundamental cellular processes in complex organisms on a genome-wide level [1]. The type of molecules are lack of tissue specificity and conserved motifs [2, 3]. Long noncoding RNAs (lncRNAs) are a class of noncoding RNAs with more than 200 nucleotides. Researches suggest that the types and number of lncRNAs are far from those of protein-coding mRNAs [4, 5]. However, only few lncRNAs have been revealed their biological functions. Aberrant expression of lncRNAs densely links with various complex diseases [6, 7], for example, hepatocellular carcinoma [8], liver cancer [9], breast cancer [10], pituitary tumors [11], coronary heart disease [12], ovarian cancer [13], Alzhermer’s diseases [14], and Huntington’s diseases [15]. Therefore, identifying the biological functions of lncRNAs helps to boost our knowledge about this class of molecules [16].

Studies demonstrate that lncRNAs regulate post-transcriptional genes, control poly-adenylation, splicing and translation by interacting with proteins [17–19]. Probing lncRNA-protein interactions (LPIs) contributes to the understanding of lncRNAs’ biological functions. Wet experiments found multiple potential LPIs. However, experimental techniques are costly and time-consuming [20, 21]. Computational methods are increasingly exploited to uncover the underlying associations between lncRNAs and proteins [19, 22, 23].

Computation-based LPI identification methods can be roughly classified into two main groups: network-based methods and supervised learning-based methods. Network-based LPI prediction methods construct a heterogeneous lncRNA-protein network and propagate the labels of LPIs on the network. Lu et al. [24] proposed a matrix multiplication-based method to score RNA-protein pairs. Li et al. [25] integrated lncRNA similarity network, protein interaction network, and LPI network and used a random walk with restart to infer LPIs. Yang et al. [26] designed a HeteSim algorithm for LPI prediction. Ge et al. [27] and Zhao et al. [21] explored two bipartite network-based LPI inference models. Zheng et al. [28] found a few LPIs based on the built multiple protein-protein similarity networks. Zhang et al. [29] used the KATZ measure to identify the linkages between lncRNAs and proteins. Hu et al. [30] proposed an eigenvalue transformation-based LPI prediction algorithm. Zhang et al. [31] exploited a linear neighborhood propagation algorithm by integrating expression profiles, interaction profiles, and sequence composition of lncRNAs and CTD features and interaction profiles of proteins. Zhao et al. [32] explored a logistic matrix factorization-based LPI discovery method combining neighborhood regularization and random walk. Zhou et al. [33] developed a Laplacian regularized least square model (LPI-SKF) to identify new interactions between lncRNAs and proteins by integrating similarity kernels. Network-based methods effectively propagated LPI labels on the heterogeneous lncRNA-protein network. However, they can not find underlying associations for an orphan protein or lncRNA.

Supervised learning methods take LPIs as positive samples and characterize LPI prediction as a binary classification problem. Muppriala et al. [34] extracted the sequence features of RNAs and proteins based on *k*-mer composition and combined SVM and random forest to predict RNA-protein associations. Wang et al. [35] took RNA-protein interactions as positive samples, randomly screened twice number of RNA-protein pairs without any association information as negative samples, and then built a naive Bayes-based prediction model. Suresh et al. [36] developed an SVM-based estimator (RPI-Pred) for RNA-protein interaction identification. Xiao et al. [37] utilized a HeteSim measure and SVM to classify interactions between lncRNAs and proteins. Deng et al. [38] selected diffusion and HeteSim features for lncRNAs and proteins and built a gradient tree boosting model (PLIPCOM) to classify each lncRNA-protein pair. Fan and Zhang [39] designed a stacked ensemble model (LPI-BLS) to infer potential new LPIs based on ensemble learning [40]. Wekesa et al. [41] proposed a categorical boosting algorithm (LPI-CatBoost) for LPI prediction.

Although supervised learning methods uncovered multiple potential associations between lncRNAs and proteins, the type of classification models are susceptible to the imbalanced ratio between positive samples and negative samples. There exists numerous unlabeled lncRNA-protein pairs and much less positive LPIs on LPI data resources. That is, the existing LPI data are severely imbalanced. More importantly, most models are evaluated on one individual LPI dataset, which may produce the prediction bias. To address the two problem, in this paper, we develop an Ensemble framework (LPI-EnEDT) with Extra tree and Decision Tree classifiers to infer new LPIs.

## Materials and methods

### Data preparation

In this study, five different LPI datasets are arranged. Dataset 1 was compiled by Li et al. [25] and contains 3479 associations between 59 proteins and 935 lncRNAs after our removing lncRNAs and proteins without any sequence information in NONCODE [42], NPInter [43], and UniProt [44] databases. Dataset 2 was built by Zheng et al. [28] and contains 3265 associations from 84 proteins and 885 lncRNAs after preprocessing similar to dataset 1. Dataset 3 was retrieved by Zhang et al. [31] and contains 4158 associations between 27 proteins and 990 lncRNAs. The three datasets are from human.

Datasets 4 and 5 provide LPI data from Arabidopsis thaliana and Zea mays, respectively. lncRNA and protein sequence information is achieved from the plant lncRNA database (PlncRNADB [45]). LPIs are downloaded at http://bis.zju.edu.cn/PlncRNADB/. The two datasets provide 948 LPIs from 35 proteins and 109 lncRNAs and 22,133 LPIs from 42 proteins and 1704 lncRNAs, respectively. Table 1 describes the details of five datasets.

**Table 1:** The statistics of LPI information

Dataset	lncRNAs	Proteins	LPIs
Dataset 1	935	59	3,479
Dataset 2	885	84	3,265
Dataset 3	990	27	4,158
Dataset 4	109	35	948
Dataset 5	1,704	42	22,133

We represent LPI network as a matrix *Y* with the element:

1 $$ {y_{{ij}}} = \left\{ {\begin{array}{*{20}{l}} {1,{\mathrm{ if lncRNAs }}{l_{i}}{\mathrm{ interacts with protein }}{p_{j}}}\\ {0,{\mathrm{ otherwise}}} \end{array}} \right.  $$

### Overview of LPI-EnEDT

**Fig. 1:**
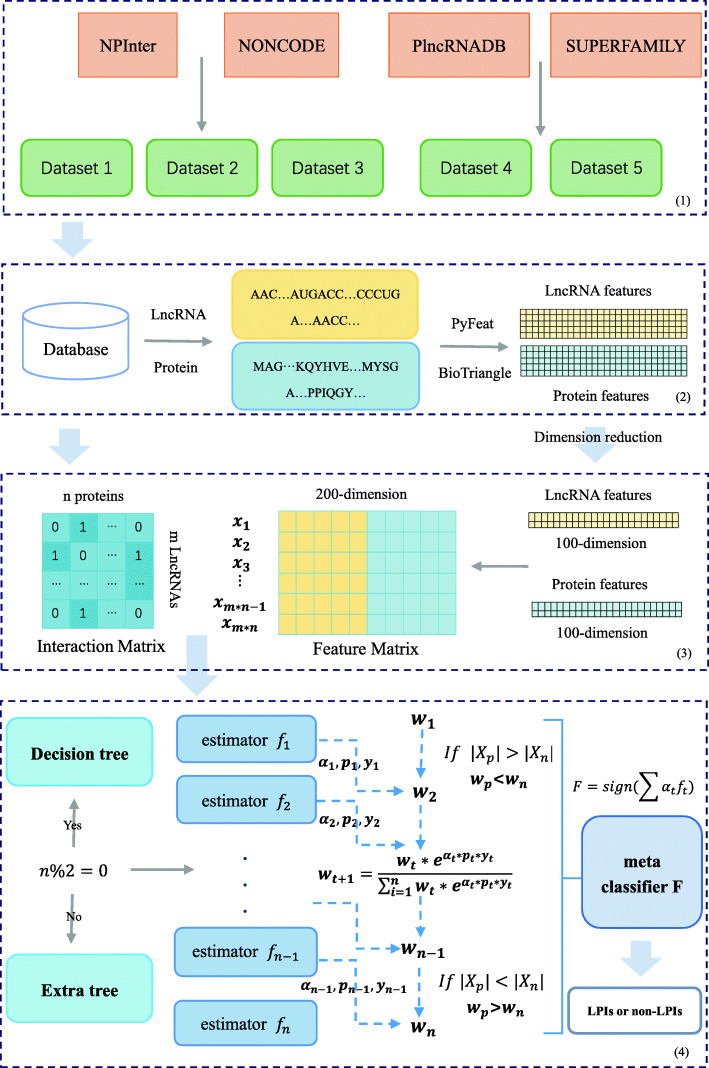
The Pipeline of the LPI-EnEDT framework. (1) Dataset arrangement. (2) Feature description. (3) Feature selection. (4) LPI classification

In this study, we develop an Ensemble framework with Extra tree and Decision Tree classifiers for imbalanced LPI data classification (LPI-EnEDT). Figure 1 depicts the pipeline of LPI-EnEDT.

As shown in Fig. 1, the LPI-EnEDT framework are grouped into four main steps: (1) Dataset arrangement. Five LPI datasets are arranged. (2) Feature description. lncRNA-protein pairs are described using Pyfeat [46] and BioTriangle [47], respectively. (3) Feature selection. The described features are reduced to *d* dimensions and concatenated as a 2*d*-dimensional vectors applied to characterize each lncRNA-protein pair. (4) LPI classification. An ensemble framework with Extra tree and decision tree classifiers is exploited to implement imbalanced LPI data classification.

### Feature selection

#### lncRNA feature selection

PyFeat [46] provides diverse features for RNA sequences. These features contain: zCurve, gcCotent, ATGC ratio and cumulative skew, Chou’s Pseudo composition, monoMonoKGap, monoDiKGap, diTriKGap, triDiKGap, triMonoKGap, monoTriKGap, diMonoKGap, and diDiKGap. We use this tool and learn a vector applied to depict each lncRNA.

#### Protein feature selection

BioTriangle [47] is a feature-rich toolkit appied to characterize proteins. These features contain amino acid composition, autocorrelation, CTD, conjoint triad, quasi-sequence order, pseudo amino acid composition. We use this toolkit and devise a vector to characterize each protein.

### Dimension reduction

The dimensions of lncRNA and protein features are decreased via principle component analysis. The reduced lncRNA and protein features are concatenated as a 2*d*-dimensional vector to denote each lncRNA-protein pair.

### LPI prediction framework

#### Problem description

There exists a few known LPIs and numerous unknown lncRNA-protein pairs on LPI datasets. The ratios of known LPIs to all lncRNA-protein pairs is 0.0631, 0.0439, 0.1556, 0.2485, and 0.3093 on the five LPI datasets, respectively. That is, existing LPI data is imbalanced. In the imbalanced LPI datasets, positive LPIs are outnumbered. To solve with the LPI data imbalanced problem, we develop an ensemble model (LPI-EnEDT) to improve the classification ability of individual classifier.

Suppose that *D*=(***X***,***Y***) denotes a known LPI dataset, where ***x***∈*X* represents a training sample characterized by an LPI feature vector of 2*d*-dimension and ***y***∈*Y* denotes the corresponding label. The proposed LPI-EnEDT framework alternately mix two weak estimators including Extra tree and decision tree classifiers to reduce the overfitting problem in the imbalanced LPI data.

#### Extra tree

The Extra tree model [48] constructs an ensemble algorithm based on unpruned regression or decision trees. It differs from other tree-based ensemble models. First, it splits nodes based on the chosen cut-points at fully random. In addition, instead of a bootstrap replica, it uses the whole learned samples to grow the trees.

Algorithm 1 describes the splitting procedure in Extra tree. In Algorithm 1, the number of attributes *K* at each node is set as $K=\sqrt {2d} $, and *n*_*min*_ denotes the minimum example size for splitting a node. Extra tree is used for a few times to construct an ensemble model. The predictions from multiple Extra trees are aggregated to generate the final prediction based on the voting method.

The explicit randomization for cut-points and attribute integration by ensemble averaging may more strongly reduce variance than the weaker randomization algorithms. To minimize the bias, instead of bootstrap replicas, Extra tree uses full original learning samples. More importantly, while ensuring the simplicity during the node splitting, Extra tree obtains much smaller constant factor than in the other ensemble-based models. The Extra tree algorithm contains three main phases:


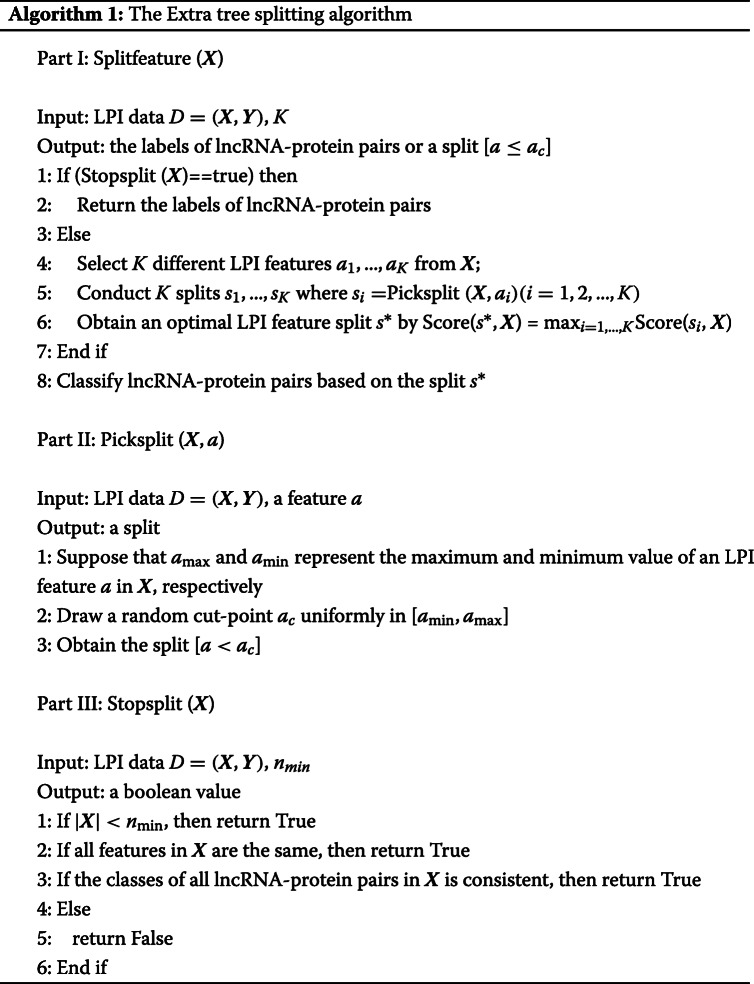


In Algorithm 1, Score(*s*^∗^,***X***) denotes the normalized Shannon information gain and can be computed by Eq. (2):

2 $$ {Score\left({s,\boldsymbol{X}}\right) = \frac{{2I\left(\boldsymbol{X}\right)}}{{{H_{c}}\left(\boldsymbol{X}\right) + {H_{s}}\left(\boldsymbol{X}\right)}}}   $$

where

3 $$ {{H_{c}}\left(\boldsymbol{X}\right) = - \sum\limits_{i = 1}^{2} {\frac{|{\boldsymbol{X}_{i}}|}{|\boldsymbol{X} |}} \times {\log_{2}}\left({\frac{|{\boldsymbol{X}_{i}}|}{|\boldsymbol{X} |}}\right)}  $$

4 $$ {{H_{s}}\left(\boldsymbol{X}\right) = - \sum\limits_{j = 1}^{2} {\frac{|{\boldsymbol{X}_{j}}|}{|\boldsymbol{X} |}} \times {\log_{2}}\left({\frac{|{\boldsymbol{X}_{j}}|}{|\boldsymbol{X} |}}\right)}  $$

5 $$ {{I(\boldsymbol{X})} = {H_{c}}\left(\boldsymbol{X}\right) - \sum\limits_{j}^{2} {\frac{|{\boldsymbol{X}_{j}}|}{|\boldsymbol{X} |} \times {H_{c}}\left({\boldsymbol{X}_{j}}\right)} }  $$

where ***X*** is the LPI sample set with the labels, *s* denotes a split where the nodes with the values smaller than the split value are put into the left on the tree; otherwise, the nodes are on the right. ***X***_*i*_ denotes two classes composed of LPIs or non-LPIs. ***X***_*j*_ denotes two sample sets on the left and right of the split node.

Extra tree has three advantages. First, each sub-decision tree in the Extra tree model uses the original dataset to train the model. Second, it randomly selects a feature to split the decision tree. Finally, it demonstrates the powerful generalization ability. Therefore, we select the Extra tree algorithm as one class of weak classifiers in the LPI-EnEDT model.

#### Decision tree

Extra tree randomly selects *K* LPI features and obtain an optimal LPI feature from the *K* features to classify lncRNA-protein pairs based on Shannon information gain. Except selecting the features used to split, other processes of decision tree are similar to Extra tree. Decision tree [49] uses a divide-and-conquer strategy to grow the trees.

Decision tree uses *gainratio* as the default splitting criterion. LPI prediction is taken as a binary classification problem. Suppose that *p*(***X***;*j*)(*j*=1,2) denotes the proportion of samples in ***X*** that belong to the *j*-th class. To measure the purity of the LPI sample set, the information entropy is defined as Eq. (6):

6 $$ Info\left(\boldsymbol{X}\right) = - \sum\limits_{j = 1}^{2} {p\left({\boldsymbol{X},j}\right)} \times {\log_{2}}\left({p\left({\boldsymbol{X},j}\right)}\right)   $$

The corresponding information gain generated by a feature *a* can be computed as Eq. (7):

7 $$ Gain\left({\boldsymbol{X},a}\right) = Info\left(\boldsymbol{X}\right) - \sum\limits_{i = 1}^{K} {\frac{{|{\boldsymbol{X}_{i}}|}}{{|\boldsymbol{X} |}}} \times Info\left({{\boldsymbol{X}_{i}}}\right)   $$

where the feature *a*(*a*∈{*a*_1_,*a*_2_,...,*a*_*k*_}) has *K* possible values for all lncRNA-protein pairs, ***X***_*i*_ denotes the sample set when *a*=*a*_*i*_. The feature with the maximum information gain is used as the splitting nodes.

#### The LPI-EnEDT method

Majority of the ensemble methods used a single weak classifier to generate the model. This may produce the prediction bias. To avoid the limitations produced by a single basic estimator and amplify the diversity, we alternately use two different predictor, that is, Extra tree and decision tree. The two basic classifier are instable and appropriate for the ensemble algorithm. Each tree produced in different ways by different algorithms can cover different subspaces, thus the combinations of multiple trees based on the ensemble algorithm can generate good classification performance.

At each iteration, LPI-EnEDT uses either extra tree or decision tree classifier as basic learners to achieve the benefits from both predictors. During learning, LPI-EnEDT evaluates each weak classifier and discards them when the estimators can not be a weak predictors or the error rate computed by them is no less than 0.5. The weak classifiers are increasingly added to the model until the performance does not improve. For each node, an feature is selected when it effectively separates the training set into multiple subsets that belong to different classes.

The weight of the *t*-th weak classifier is computed to measure its importance among all weak classification models by Eq. (8):

8 $$ \alpha_{t} = \frac{1}{2}{\log_{e}}\frac{{1 - error(f_{t})}}{{error(f_{t})}}   $$

In LPI dataset, there are a few positive samples (LPIs) and numerous unknown lncRNA-protein pairs, which result in the problem of sample imbalance. During the process of selecting a weak classifier, to solve the imbalanced LPI data, for each lncRNA-protein pair ***x***_*i*_, we update its weight at the (*t*+1)-th iteration as Eq. (9):

9 $$ {\boldsymbol{w}_{t + 1}} = \frac{{{\boldsymbol{w}_{t}} * {e^{{\alpha_{t}}*{\boldsymbol{p}_{t}}*{\boldsymbol{y}_{t}}}}}}{{{\sum\nolimits}_{i = 1}^{n} {{\boldsymbol{w}_{t}} * {e^{{\alpha_{t}}*{\boldsymbol{p}_{t}}*{\boldsymbol{y}_{t}}}}} }}   $$

where ***p***_*t*_ and ***y***_*t*_ denote the predicted labels and real labels at the *t*-th iteration, respectively. Based on the classification results at the last iteration, LPI-EnEDT assigns a higher weight to a class with minor samples to reduce the affect produced by the imbalanced LPI data.

After *t* iterations, the meta classifier can be built by Eq. (10):

10 $$ F(t)=sign(\sum {{\alpha_{t}}} {f_{t}})   $$

At each odd number of iteration, the most appropriate Extra tree is selected as a weak classifier. At each even number of iteration, the most appropriate decision tree is selected as a weak classifier. Based on the meta classifier *F*_*t*_ learned through *t* iterations, the performance *c*_*t*_ of the LPI-EnEDT model on the testing set is computed. Compared to the iteration where the model obtains the best performance *p*_*best*_, if the performance at the *t*-th iteration is improved or keeps instable, *f*_*t*_ will be added to the final classification model *F*. After *t* iteration, the model *F*_*best*_, composed of *t* weak classifiers, computes the best performance. For the following *M* iterations, if the performance at each iteration does not improve, the iteration will be stopped and *F*_*best*_ will be selected as the final classification model. Algorithm 2 describes the LPI classification process based on LPI-EnEDT.


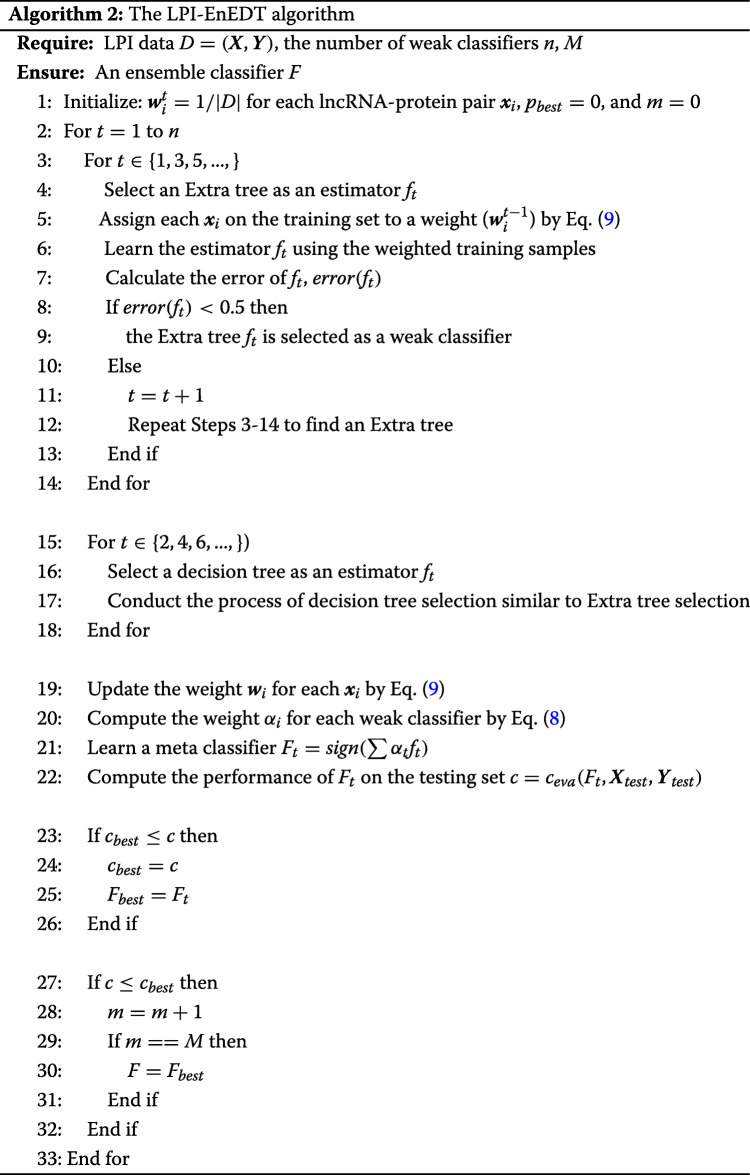


## Results

### Evaluation metrics

Six evaluation metrics are applied to measure the proposed LPI-EnEDT framework: precision, recall, accuracy, F1-score, AUC and AUPR. They are defined as follows:

11 $$ Precision=\frac{{TP}}{{TP + FP}}  $$

12 $$ Recall=\frac{{TP}}{{TP+FN}}  $$

13 $$ Accuracy=\frac{{TP+TN}}{{TP+TN+FP+FN}}  $$

14 $$ F1-score=\frac{{2TP}}{{2TP+FP+FN}}  $$

where TP, TN, FP, and FN indicates true positive, true negative, false positive, and false negative, respectively. AUC and AUPR are the areas under the receiver operating characteristic (ROC) curve and Precision-Recall (PR) curve, respectively. For the six metrics, higher values demonstrate better performance.

The experiments are repeated for 20 times and the final results are computed by averaging the 20 round performance. In Algorithm 2, *c*_*eva*_(*F*_*t*_,***X***_*test*_,***Y***_*test*_) is computed according to the AUC value because when AUCs obtained from LPI-EnEDT are higher, other five measurements are still better.

### Experimental settings

The parameters in Pyfeat is set as: kGap = 5, opti-mumDataset = 1, kTuple = 3, pseudoKNC = 0, gcContent = 1, zCurve = 1, cumulativeSkew = 1, monoTri = 1, monoMono = 1, diMono = 1, monoDi = 1, triMono = 1, atgcRatio = 1, triDi = 1, diTri = 1, diDi = 1. The parameters in BioTriangle and LPI-SKF are set as the defaults provided by Dong et al. [47] and Zhou et al. [33], respectively. The parameters in other LPI prediction algorithms are set the corresponding values shown in Table 2.

**Table 2:** Parameter Settings

Method	Parameter setting
LPI-BLS	s=1, c= 10^−10^, N1=3, N2=60, N3=900
LPI-CastBoost	learning_rate=0.5, loss_function=’Logloss’
	logging_level=’Verbose’
PLIPCOM	learning_rate=0.01,n_estimators=100
	min_samples_split=2, max_depth=3
LPI-EnEDT	n_estimators=10, depth=5, split=5, neighbours=3

We conduct grid search and observe that when *d*=100, LPI-EnEDT computes the best measurements. Therefore, we construct two 100-dimensional vectors applied to lncRNA and protein feature description. Three 5-fold Cross Validations (CVs) on lncRNAs, proteins and lncRNA-protein pairs are conducted to evaluate the performance of LPI-EnEDT.

1. 5-fold CV on lncRNAs (*CV*_*l*_, LPI prediction for new lncRNAs): 80% of lncRNAs are randomly selected as a training set and the remaining 20% is taken as a testing set in each round.
2. 5-fold CV on proteins (*CV*_*p*_, LPI prediction for new proteins): 80% of proteins are randomly selected as a training set and the remaining 20% is taken as a testing set in each round.
3. 5-fold CV on lncRNA-protein pairs (*CV*_*lp*_, LPI prediction for lncRNA-protein pairs): 80% of lncRNA-protein pairs are randomly selected as training set and the remaining 20% is taken as a testing set in each round.

In addition, known LPI datasets are unbalanced. Therefore, we develop an ensemble learning model for imbalanced data, LPI-EnEDT. In the experiments, the ratio of positive samples to negative samples is randomly selected to solve the problem of imbalanced LPI data classification.

### Comparison with four state-of-the-art LPI prediction methods

We compare the proposed LPI-EnEDT algorithm with four state-of-the-art LPI discovery models to measure the classification ability for imbalanced LPI data, that is, LPI-BLS, LPI-CatBoost, PLIPCOM, and LPI-SKF. LPI-BLS, LPI-CatBoost, and PLIPCOM are three supervised learning-based LPI identification techniques and LPI-SKF is a network-based inference approach.

**Fig. 2:**
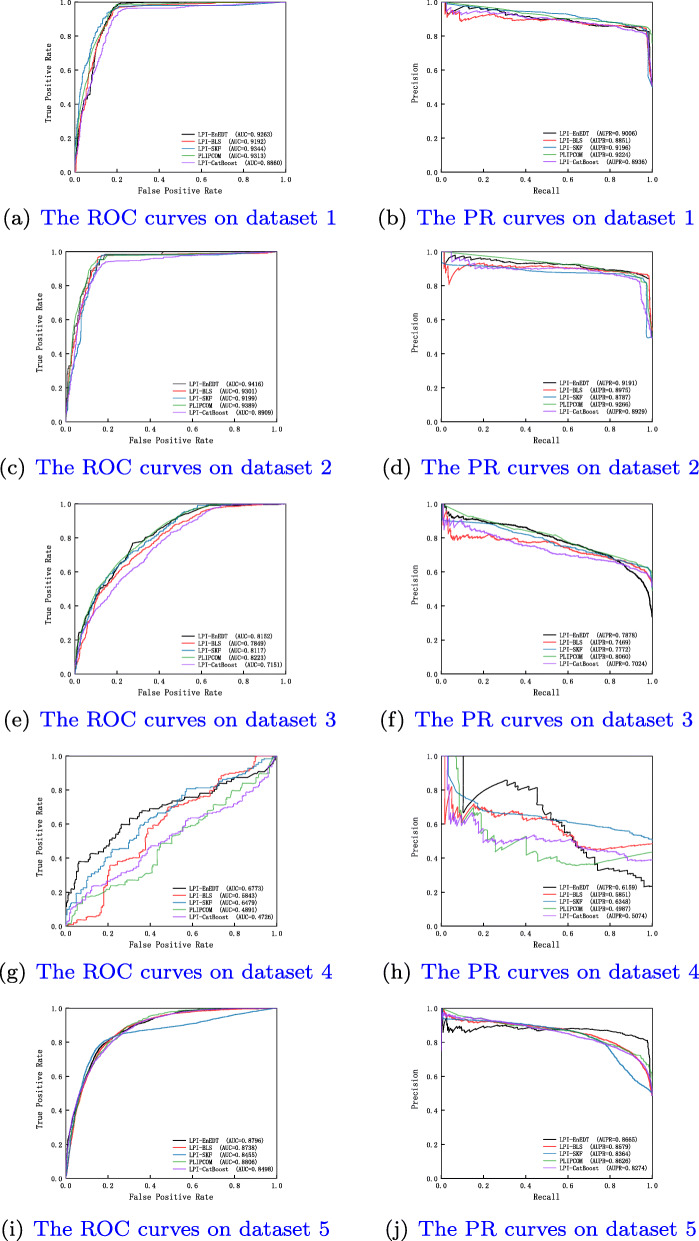
The ROC curves and the PR curves of different methods under *CV*_*l*_

Table 1 in the Supplementary Materials lists the results from five LPI prediction algorithms under *CV*_*l*_. As shown in Table 1 in the Supplementary Materials, LPI-EnEDT calculates the best average recall, accuracy, F1-score, AUC, and AUPR on the five datasets, are much better than LPI-BLS, LPI-CatBoost, PLIPCOM, and LPI-SKF. For example, LPI-EnEDT computes the highest AUC values on all datasets and obtains the best average AUC of 0.8480, which is better 3.48%, 10.04%, 4.20%, and 1.90% than LPI-BLS, LPI-CatBoost, PLIPCOM, and LPI-SKF, respectively. More importantly, LPI-EnEDT calculates the optimal average AUPR value of 0.8175, better 2.81%, 6.46%, 2.12%, and 1.00% than the four methods. Figure 2 shows the ROC and PR curves of all five algorithms on five datasets under *CV*_*l*_. The results demonstrate that LPI-EnEDT can more accurately infer underlying proteins for a new lncRNA.

**Fig. 3:**
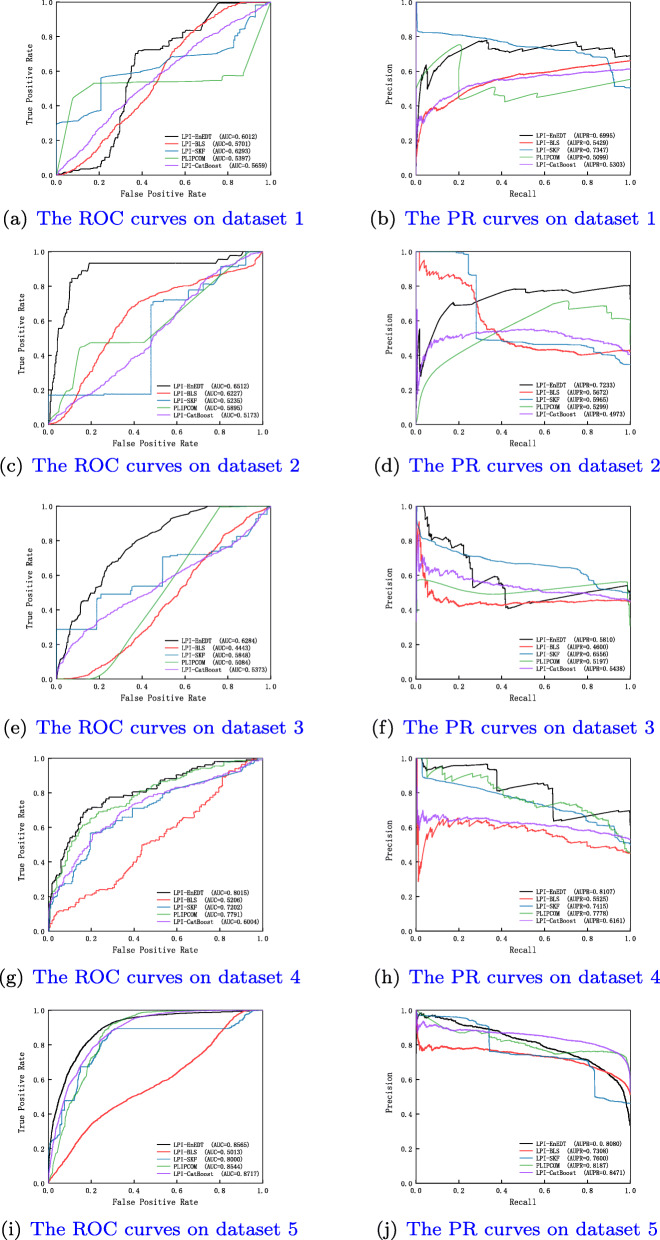
The ROC curves and the PR curves under *CV*_*p*_

Table 2 in the Supplementary Materials describes the experimental results under *CV*_*p*_. It can be analyzed that LPI-EnEDT computes the best average recall of 0.8311, accuracy of 0.6626, F1-score of 0.6700, AUC of 0.7078 and AUPR of 0.7265 on the five datasets. In particular, LPI-EnEDT obtains the best recalls on all datasets. More importantly, LPI-EnEDT calculates the best AUCs on datasets 2-4, the best AUPRs on datasets 2 and 4, demonstrating the powerful LPI prediction ability of LPI-EnEDT on datasets 2 and 4. Fig. 3 describes the ROC and PR curves of five LPI prediction algorithms under *CV*_*p*_. In general, LPI-EnEDT is appropriate for finding interacting lncRNAs with a new protein.

**Fig. 4:**
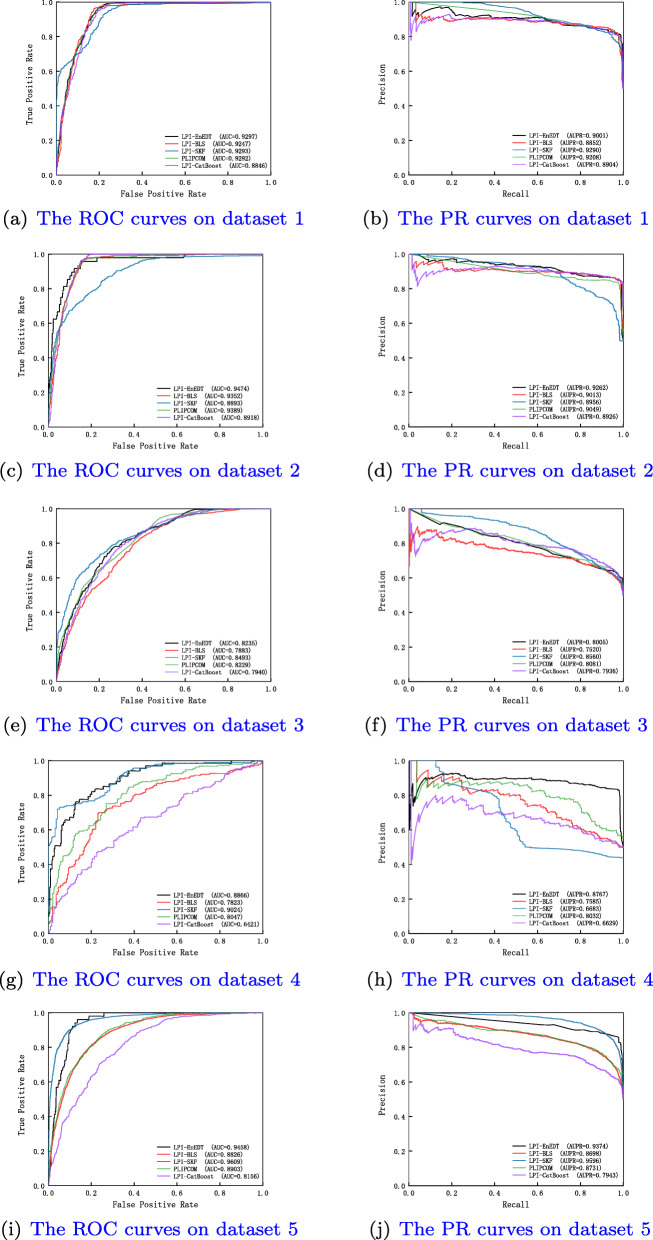
The ROC curves and the PR curves of different methods under *CV*_*lp*_

The performance under *CV*_*lp*_ are showed in Table 3 in the Supplementary Materials. From Table 3 in the Supplementary Materials, we can examine that LPI-EnEDT obtains the best performance. In particular, precision, accuracy, F1-score, and AUPR computed by LPI-EnEDT are much better than other four representative LPI identification algorithms. For example, LPI-EnEDT investigates the highest average F1-score of 0.8420, which is 3.00% better than the second-best method (PLIPCOM) and 10.68% than the third-best method (LPI-CatBoost). The average AUPR value from LPI-ENEDT outperforms 2.95% and 2.98% than the second-best and the third-best models (PLIPCOM and LPI-SKF). Figure 4 depicts the ROC and PR curves of five LPI identification models under *CV*_*lp*_. The results manifest the superior learning ability of LPI-EnEDT. In addition, we notice that LPI-EnEDT has the optimal classification performance on dataset 2 under the three CVs. The comparative results again display that LPI-EnEDT helps to boost the LPI classification ability and uncover new LPIs from the observations.

### Case study

In this section, we further reveal possible association information by case analyses.

#### Finding associated proteins for new lncRNAs

LINC00294 is an lncRNA highly expressed in normal brain tissues and distinctly downregulated in glioma cell lines and GBM tissues. Its overexpression may prevent glioma cell proliferation but enhance apoptosis. More importantly, NEFM, a tumor suppressor, was reported to be significantly reduced in cancerous conditions and boost in glioma cells through LINC00294 up-regulation [50]. LINC00294 induced by GRP78 may still promote the progression of cervical cancer [51]. In addition, Bak-IIIa may improve LPS-induced inflammatory damage in HUVECs through upregulating LINC00294 [52].

To predict new associated proteins for LINC00294, all its interaction data are masked. The five LPI prediction models are then applied to predict the underlying associations between LINC00294 and proteins. The discovered top 5 proteins associated with LINC00294 are described in Table 3. It can be found that Q13148 and P35637 have been inferred to interact with LINC00294 in dataset 3. Although the interactions between Q13148 and P35637 and LINC00294 are unknown in dataset 3, they have been reported to related to LINC00294 in datasets 1 and 2. The results again confirm the classification performance of LPI-EnEDT. Therefore, LPI-EnEDT is appropriate to interacting protein prediction for a new lncRNA.

**Table 3:** The predicted top 5 proteins interacting with LINC00294

Dataset	Proteins	Confirmed	LPI-EnEDT	LPI-BLS	LPI-CatBoost	LPI-SKF	PLIPCOM
Dataset 1	O00425	YES	1	6	4	9	3
	Q15717	YES	2	1	1	8	1
	Q9Y6M1	YES	3	3	2	1	5
	P35637	YES	4	2	9	3	8
	Q9NZI8	YES	5	5	5	2	2
Dataset 2	Q15717	YES	1	2	3	9	10
	Q9NZI8	YES	2	4	9	2	3
	Q9Y6M1	YES	3	1	1	3	1
	P35637	YES	4	3	7	1	9
	P31483	YES	5	13	5	5	15
Dataset 3	O00425	YES	1	1	4	2	2
	Q9NUL5	YES	2	13	1	4	4
	Q9Y6M1	YES	3	4	3	1	3
	Q13148	NO	4	5	10	14	11
	P35637	NO	5	9	5	9	7

#### Finding potential lncRNAs interacting with new proteins

Q9Y6M1 is RNA-binding protein. The protein can recruit target transcripts to cytoplasmic mRNPs. The transcript ‘caging’ into mRNPs can promote the transport and transient storage of mRNA. It can also modulate the rate and location where target transcripts encounter the translational apparatus and protect them from microRNA-mediated degradation or endonuclease attacks [53]. It can still discover novel autoimmune peptide epitopes of prostein in prostate Cancer [54].

Q9Y6M1 associates with 364, 342, and 387 lncRNAs on the three human datasets, respectively. We mask all interaction data for Q9Y6M1 and utilize the proposed LPI-EnEDT framework to predict lncRNA candidates related to the protein. The top 5 lncRNAs with the highest interaction probabilities with Q9Y6M1 are listed in Table 4. Although the predicted interactions between H19 and Q9Y6M1 are unknown in datasets 1and 2, the interaction can be found in dataset 3.

**Table 4:** The predicted top 5 lncRNAs interacting with Q9Y6M1

Dataset	lncRNAs	Confirmed	LPI-EnEDT	LPI-BLS	LPI-CatBoost	LPI-SKF	PLIPCOM
Dataset 1	H19	NO	1	735	515	730	516
	XIST	YES	2	247	328	246	328
	SNHG1	YES	3	686	494	322	495
	NONHSAG073380	YES	4	361	736	185	736
	SLC2A3P1	YES	5	799	700	394	700
Dataset 2	n343060	YES	1	685	4	750	4
	HOTTIP	NO	2	267	66	801	66
	H19	NO	3	558	107	149	107
	n385725	YES	4	261	12	107	12
	SNHG1	YES	5	29	16	508	16
Dataset 3	LINC00638	YES	1	767	851	340	822
	NONHSAG038845	YES	2	764	836	118	805
	PTENP1	YES	3	647	292	161	217
	NONHSAG048098	YES	4	986	504	11	242
	NONHSAG058184	YES	5	969	210	777	370

In addition, we predict that the lncRNA HOTTIP may interact with Q9Y6M1 ranked as 2 on dataset 2. HOTTIP has dense linkages with a few disease. For example, it can promote the proliferation, survival and migration of pancreatic cancer cells [55]. Its overexpression may enhance chemoresistance of osteosarcoma cell [56]. It is also used as possible diagnostic and prognostic biomarker for gastric cancer [57]. In dataset 2, there are 885 lncRNAs possibly associated with Q9Y6M1 and the interaction between HOTTIP and Q9Y6M1 is ranked as 2, 267, 66, 801, and 66 by LPI-EnEDT, LPI-BLS, LPI-CatBoost, LPI-SKF, and PLIPCOM, respectively. Therefore, we predict that HOTTIP may interact with Q9Y6M1 and the interaction needs further biomedical experimental validation. In general, LPI-EnEDT can be used to LPI prediction for a new protein.

#### Finding new LPIs based on known LPIs

**Fig. 5:**
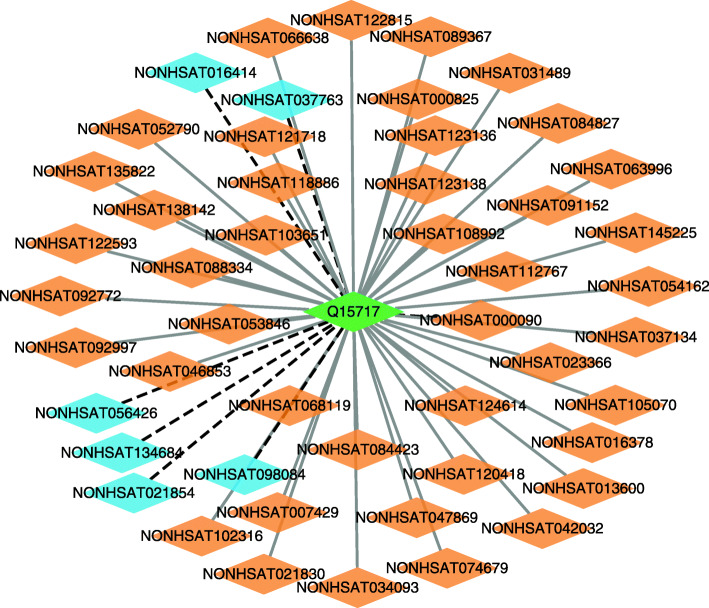
The predicted top 50 LPIs on dataset 1

**Fig. 6:**
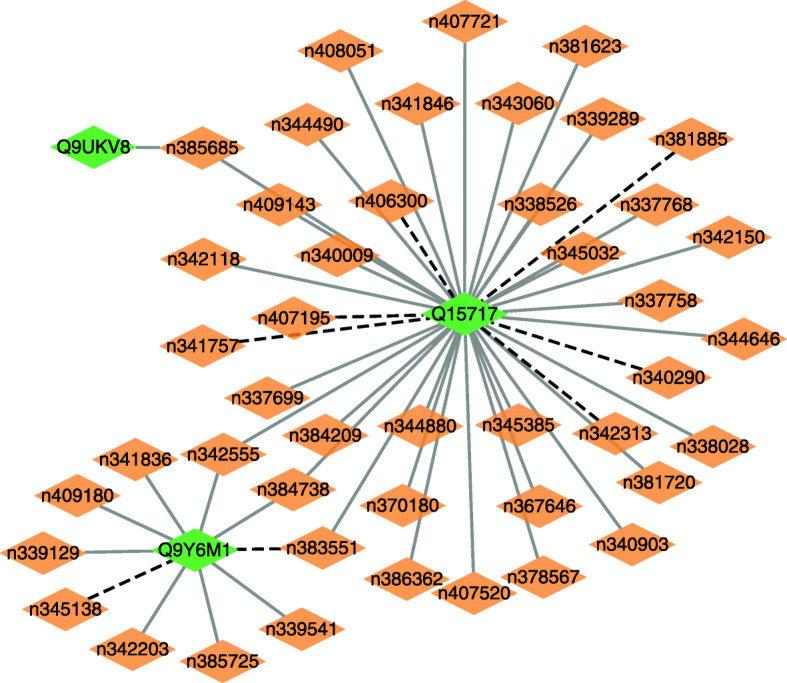
The predicted top 50 LPIs on dataset 2

**Fig. 7:**
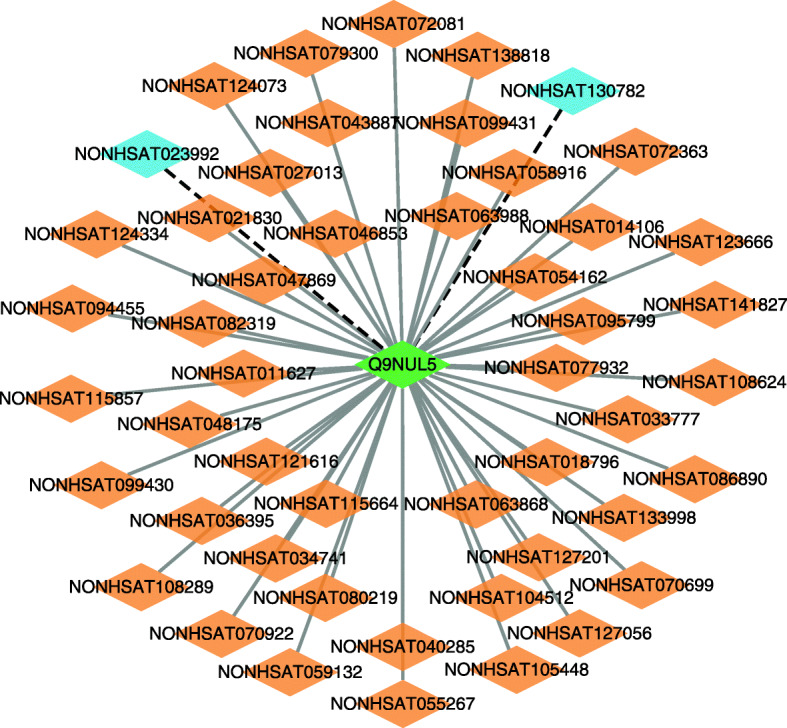
The predicted top 50 LPIs on dataset 3

**Fig. 8:**
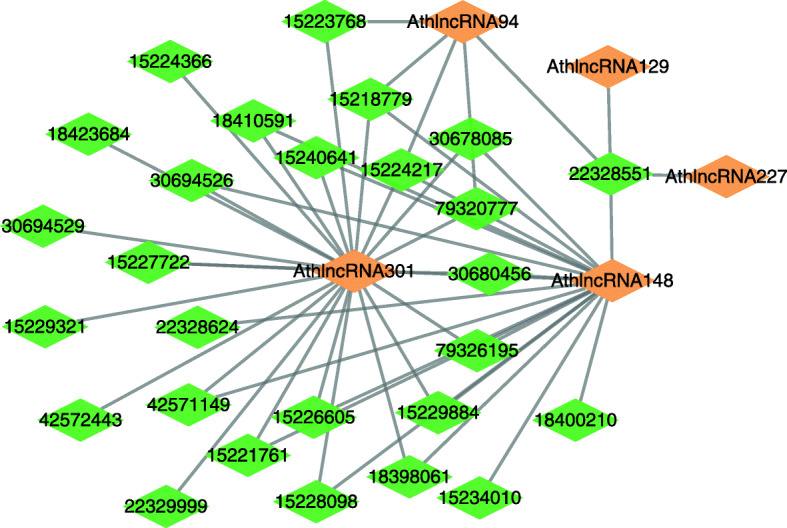
The predicted top 50 LPIs on dataset 4

**Fig. 9:**
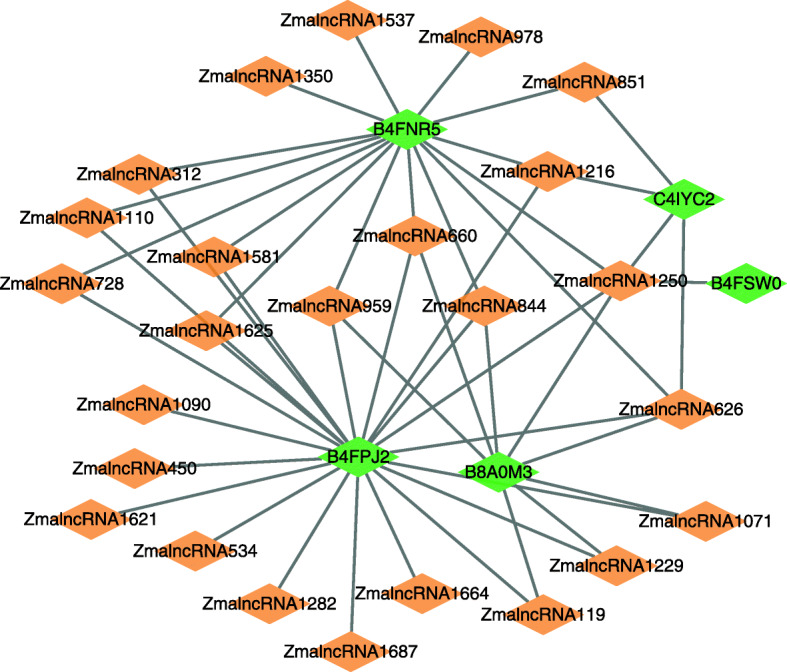
The predicted top 50 LPIs on dataset 5

We further infer possible association information between lncRNAs and proteins based on LPI-EnEDT. We compute the interaction probabilities for each lncRNA-protein pair. The inferred top 50 LPIs, including known LPIs and unlabeled lncRNA-protein pairs, are shown in Figs. 5, 6, 7, 8 and 9. In the five figures, gray solid and black dotted lines denote labeled LPIs and unlabeled lncRNA-protein pairs inferred by LPI-EnEDT, respectively. Lime green diamonds denote proteins. Dark orange and deep sky blue diamonds describe lncRNAs whose interactions with given proteins are known and unknown, respectively.

It can be observed that the associations between NRON and Q15717, LINC00958 and Q9Y6M1, RP11-819C21.1 and Q9NUL5, AthlncRNA32 and 22328551, and ZmalncRNA1113 and B4FPJ2 have the highest association scores among unlabeled lncRNA-protein pairs on datasets 1-5, respectively. On the five datasets, there are separately 55,165, 74,340, 26,730, 3,815, and 71,568 lncRNA-protein pairs and the predicted top 5 LPIs are ranked as 1, 21, 7, 185, and 346, respectively.

The lncRNA NRON can regulate osteoclastogenesis during orthodontic bone resorption [58], reduce atrial fibrosis by promoting NFATc3 phosphorylation [59], inhibit breast cancer development via regulating miR-302b/SRSF2 axis [60]. More importantly, its dysregulation in diabetic cardiomyopathy can prevent the injury and inflammation induced by high glucose cardiomyocyte [61]. Q15717 is a RNA-binding protein. The protein is involved in the differentiation of embryonic stem cells. It can enhance the stability of the leptin mRNA [62], regulate the p53/TP53 expression, and mediate the CDKN2A anti-proliferative activity. In dataset 2, there are 55,165 possible lncRNA-protein pairs. Among the 55,165 lncRNA-protein pairs, the interaction between NRON and Q15717 is ranked as 1. Therefore, we infer that NRON may associate with Q15717 and need further validation. Although the interaction data has not been confirmed, we hope to further validate it through biomedical experiments.

## Discussion and further research

The identification of new LPIs based on computational approaches contributes to understanding the biological functions and mechanisms of lncRNAs. However, there are few LPI data and a vast of unlabeled lncRNA-protein pairs. That is, existing LPI datasets are severely imbalanced. Therefore, it is a challenging task to build a classification model to alleviate LPI class imbalanced problem.

To address the above problem, in this study, an ensemble model (LPI-EnEDT) with two types of weak classifiers is designed to classify unknown lncRNA-protein pairs. First, LPI-EnEDT arranges five LPI datasets. Second, it constructs a feature vector to characterize lncRNA-protein pairs based on the existing bioinformatics tools and the dimensional reduction method. Finally, it integrates Extra tree and decision tree classifiers and develops an ensemble framework combining multiple weak classifiers to identify LPI candidates.

Unlike the other boosting methods, our proposed LPI-EnEDT model alternately uses two different estimators, extra tree and decision tree. At each odd number of iterations, the best Extra tree is selected as a weak predictor. At each even number of iterations, the best decision tree is selected as a weak predictor. The number of two basic classifiers is the same, and the weight of each weak classifier is determined by its loss value. The parameter settings in the two classifiers are still the same. By this way, we take advantages of two basic predictors while avoiding the limitations produced by using a single basic classifier.

LPI-EnEDT is compared to LPI-BLS, LPI-CatBoost, LPI-SKF, and PLIPCOM. It computes the best average AUC and AUPR on five LPI datasets under the three CVs. The comparative results demonstrate the superior classification performance of the proposed LPI-EnEDT model. During LPI prediction, network-based methods use the entire LPI matrix to train a model, while machine learning-based methods only use a fraction of LPI data to learn a model. The abundance of data may affect the prediction performance of models, resulting in that network-based methods obtain better performance than machine learning-based methods in a few cases. However, network-based methods have one limitation: they can not find possible LPI for an orphan lncRNA or protein. Therefore, machine learning-based methods may be more appropriate for LPI prediction. In addition, In five LPI datasets, the number of proteins is 59, 84, 27, 35, and 42, respectively. Under *CV*_*p*_, samples are relatively smaller, and thus the performance is generally low.

The LPI-EnEDT algorithm computes the best LPI prediction ability. The reason may be that LPI-EnEDT alternately integrates Extra tree and decision tree classifiers instead of using a simple weak classifier. Both Extra tree and decision tree classifiers have individual characteristics and weaknesses. Ensemble learning helps LPI-EnEDT fully utilize both estimator’s advantages and discard their individual limitations. In the future, we will exploit better ensemblemodel applied to LPI classification by combining various types of weak classifiers.

## Supplementary Information

**Additional file 1** Supplementary Material.

## Data Availability

Source codes and datasets are freely available for download at https://github.com/plhhnu/LPI-EnEDT.

## Abbreviations

LPI-EnEDT: An Ensemble Framework with Extra Tree and Decision Tree Classifiers
LPI: Long noncoding RNA-Protein Interaction
Extra Tree: Extremely Randomized Trees
lncRNAs: Long noncoding RNAs
CVs: Cross Validations
*CV*_*l*_: Cross Validation on lncRNAs
*CV*_*p*_: Cross Validation on proteins
*CV*_*lp*_: Cross Validation on lncRNA-protein pairs
