# Supplementary material for: LPI-EnEDT: an ensemble framework with extra tree and decision tree classifiers for imbalanced lncRNA-protein interaction data classification
Source: BioData Min. 2021 Dec 3;14:50. doi: 10.1186/s13040-021-00277-4 (PMC8642957; doi:10.1186/s13040-021-00277-4)
Supplement: Supplementary file 1 — Additional file 1 Supplementary Material. [file 13040_2021_277_MOESM1_ESM.pdf]

Table 1 The performance of five LPI prediction methods on CV_l

Metric	Dataset	LPI-BLS	LPI-CatBoost	PLIPCOM	LPI-SKF	LPI-EnEDT
Precision	Dataset 1	0.8458±0.0014	0.8317±0.0132	0.8428±0.0060	**0.8757±0.0086**	0.8022±0.0145
	Dataset 2	0.8547±0.0031	0.8220±0.0139	0.8537±0.0065	**0.8627±0.0223**	0.8446±0.0203
	Dataset 3	0.7110±0.0011	0.6871±0.0060	0.7173±0.0084	**0.7298±0.0153**	0.6640±0.0163
	Dataset 4	0.5653±0.0088	0.4613±0.0369	0.4894±0.0508	**0.6108±0.0249**	0.5224±0.0917
	Dataset 5	**0.7901±0.0021**	0.7713±0.0040	0.7721±0.0021	0.7517±0.0098	0.7092±0.0498
	Ave.	0.7534	0.7147	0.7351	**0.7661**	0.7085
Recall	Dataset 1	0.6550±0.0009	0.8331±0.0140	0.9632±0.0028	0.5932±0.0156	**0.9689±0.0096**
	Dataset 2	0.6738±0.0013	0.8399±0.0201	0.9628±0.0043	0.5212±0.0107	**0.9737±0.0200**
	Dataset 3	0.6270±0.0006	0.6154±0.0241	0.7618±0.0141	0.6226±0.0058	**0.8432±0.0235**
	Dataset 4	0.5328±0.0074	0.3539±0.0700	0.3190±0.0668	**0.6056±0.0280**	0.5921±0.0899
	Dataset 5	0.7063±0.0038	0.7921±0.0135	0.8569±0.0037	0.6727±0.0037	**0.9015±0.0184**
	Ave.	0.6390	0.6869	0.7727	0.6030	**0.8560**
Accuracy	Dataset 1	0.7512±0.0005	0.8310±0.0071	**0.8917±0.0039**	0.7254±0.0032	0.8650±0.0073
	Dataset 2	0.7620±0.0018	0.8258±0.0064	**0.8987±0.0034**	0.7065±0.0081	0.8973±0.0134
	Dataset 3	0.6605±0.0012	0.6677±0.0091	**0.7298±0.0034**	0.6544±0.0092	0.7080±0.0097
	Dataset 4	0.5424±0.0048	0.4801±0.0201	0.4972±0.0306	0.5727±0.0196	**0.5993±0.0759**
	Dataset 5	0.7337±0.0025	0.7785±0.0067	**0.8018±0.0018**	0.6726±0.0036	0.7665±0.0266
	Ave.	0.6900	0.7166	0.7638	0.6663	**0.7672**
F1-score	Dataset 1	0.7381±0.0012	0.8314±0.0067	**0.8989±0.0033**	0.6298±0.0070	0.8775±0.0077
	Dataset 2	0.7533±0.0020	0.8282±0.0067	0.9048±0.0027	0.5828±0.0117	**0.9053±0.0131**
	Dataset 3	0.6663±0.0008	0.6480±0.0148	0.7377±0.0034	0.5950±0.0086	**0.7426±0.0108**
	Dataset 4	0.5483±0.0081	0.3812±0.0573	0.3783±0.0597	0.5401±0.0232	**0.6552±0.0639**
	Dataset 5	0.7458±0.0030	0.7812±0.0080	**0.8121±0.0018**	0.6345±0.0041	0.7862±0.0298
	Ave.	0.6904	0.6940	0.7464	0.5964	**0.7932**
AUC	Dataset 1	0.9192±0.0005	0.8860±0.0048	0.9313±0.0030	**0.9344±0.0073**	0.9263±0.0073
	Dataset 2	0.9301±0.0017	0.8909±0.0044	0.9389±0.0034	0.9199±0.0149	**0.9416±0.0134**
	Dataset 3	0.7849±0.0020	0.7151±0.0112	**0.8223±0.0029**	0.8117±0.0159	0.8152±0.0097
	Dataset 4	0.5843±0.0094	0.4726±0.0270	0.4891±0.0326	0.6479±0.0379	**0.6773±0.0759**
	Dataset 5	0.8738±0.0028	0.8498±0.0064	**0.8806±0.0019**	0.8455±0.0076	0.8796±0.0266
	Ave.	0.8185	0.7629	0.8124	0.8319	**0.8480**
AUPR	Dataset 1	0.8851±0.0022	0.8936±0.0049	**0.9224±0.0037**	0.9196±0.0092	0.9006±0.0077
	Dataset 2	0.8975±0.0032	0.8929±0.0050	**0.9266±0.0044**	0.8787±0.0260	0.9191±0.0087
	Dataset 3	0.7469±0.0006	0.7024±0.0109	**0.8060±0.0044**	0.7772±0.0198	0.7878±0.0226
	Dataset 4	0.5851±0.0109	0.5074±0.0254	0.4987±0.0272	**0.6348±0.0340**	0.6159±0.1024
	Dataset 5	0.8579±0.0036	0.8274±0.0079	0.8626±0.0027	0.8364±0.0170	**0.8665±0.0123**
	Ave.	0.7945	0.7647	0.8033	0.8093	**0.8175**

Table 2 The performance of five LPI prediction methods on CV_p

Metric	Dataset	LPI-BLS	LPI-CatBoost	PLIPCOM	LPI-SKF	LPI-EnEDT
Precision	Dataset 1	0.5370±0.0347	0.3405±0.1562	0.3541±0.1209	**0.7009±0.1208**	0.6062±0.2160
	Dataset 2	0.5769±0.0287	0.3468±0.1536	0.3879±0.1793	0.6138±0.1316	**0.6558±0.1912**
	Dataset 3	0.4479±0.0234	0.5419±0.0476	0.3772±0.1050	**0.6639±0.1119**	0.5203±0.1368
	Dataset 4	0.5319±0.0042	0.6023±0.0286	**0.7413±0.0151**	0.7261±0.0412	0.6173±0.0619
	Dataset 5	0.4164±0.0122	**0.7868±0.0085**	0.7459±0.0037	0.7264±0.1465	0.7156±0.0645
	Ave.	0.5020	0.5237	0.5213	**0.6862**	0.6211
Recall	Dataset 1	0.5264±0.0130	0.2567±0.1423	0.2165±0.0725	0.5415±0.0702	**0.7846±0.2932**
	Dataset 2	0.5486±0.0204	0.2325±0.1309	0.1744±0.1197	0.4114±0.0551	**0.7990±0.2857**
	Dataset 3	0.4819±0.0104	0.3637±0.0817	0.3023±0.1209	0.4982±0.0746	**0.7680±0.2149**
	Dataset 4	0.5479±0.0042	0.5278±0.0600	0.6730±0.0125	0.5402±0.0415	**0.8742±0.0575**
	Dataset 5	0.7993±0.0470	0.8122±0.0338	0.8473±0.0155	0.5811±0.0589	**0.9295±0.0467**
	Ave.	0.5808	0.4386	0.4427	0.5145	**0.8311**
Accuracy	Dataset 1	0.5382±0.0252	0.5204±0.0694	0.5173±0.0424	0.5867±0.0757	**0.6781±0.1261**
	Dataset 2	0.5672±0.0181	0.5092±0.0641	0.5298±0.0562	0.5220±0.0482	**0.6862±0.1274**
	Dataset 3	0.4708±0.0139	0.5361±0.0321	0.4899±0.0349	**0.5584±0.0777**	0.5246±0.1091
	Dataset 4	0.5135±0.0038	0.5767±0.0126	**0.7172±0.0109**	0.6202±0.0332	0.6638±0.0469
	Dataset 5	0.5089±0.0004	**0.7951±0.0141**	0.7785±0.0051	0.6636±0.0644	0.7605±0.0433
	Ave.	0.5197	0.5875	0.6065	0.5902	**0.6626**
F1-score	Dataset 1	0.5285±0.0228	0.2567±0.1423	0.2494±0.0853	0.5399±0.0745	**0.6075±0.2119**
	Dataset 2	0.5617±0.0246	0.2622±0.1347	0.2131±0.1301	0.4092±0.0634	**0.5923±0.2158**
	Dataset 3	0.4635±0.0172	0.4175±0.0750	0.3144±0.1120	0.4929±0.0804	**0.5882±0.1334**
	Dataset 4	0.5372±0.0005	0.5389±0.0305	0.7030±0.0103	0.5468±0.0408	**0.7490±0.0389**
	Dataset 5	0.5467±0.0250	**0.7970±0.0184**	0.7920±0.0071	0.5908±0.0734	0.7930±0.0425
	Ave.	0.5275	0.4545	0.4544	0.5159	**0.6700**
AUC	Dataset 1	0.5701±0.0508	0.5659±0.0734	0.5397±0.0855	**0.6293±0.1142**	0.6012±0.1261
	Dataset 2	0.6227±0.0328	0.5173±0.0987	0.5895±0.0743	0.5235±0.0899	**0.6512±0.1274**
	Dataset 3	0.4443±0.0269	0.5373±0.0421	0.5084±0.0512	0.5848±0.1577	**0.6284±0.1091**
	Dataset 4	0.5206±0.0088	0.6004±0.0148	0.7791±0.0124	0.7202±0.0571	**0.8015±0.0469**
	Dataset 5	0.5013±0.0025	**0.8717±0.0133**	0.8544±0.0063	0.8000±0.1136	0.8568±0.0433
	Ave.	0.5318	0.6185	0.6542	0.6516	**0.7078**
AUPR	Dataset 1	0.5429±0.0415	0.5303±0.0744	0.5099±0.0686	**0.7347±0.1155**	0.6995±0.1989
	Dataset 2	0.5672±0.0181	0.4973±0.0760	0.5299±0.0719	0.5965±0.1215	**0.7233±0.1710**
	Dataset 3	0.4600±0.0243	0.5438±0.0333	0.5197±0.0420	**0.6556±0.1277**	0.5810±0.1505
	Dataset 4	0.5525±0.0034	0.6161±0.0211	0.7778±0.0168	0.7415±0.0543	**0.8107±0.0413**
	Dataset 5	0.7308±0.0046	**0.8471±0.0164**	0.8187±0.0119	0.7600±0.1657	0.8080±0.0524
	Ave.	0.5707	0.6069	0.6312	0.6977	**0.7265**

Table 3 The performance of five LPI prediction methods on CV_{lp}

Metric	Dataset	LPI-BLS	LPI-CatBoost	PLIPCOM	LPI-SKF	LPI-EnEDT
Precision	Dataset 1	**0.8539±0.0012**	0.8340±0.0170	0.8440±0.0045	0.7979±0.0337	0.8472±0.0149
	Dataset 2	0.8668±0.0018	0.8191±0.0224	0.8478±0.0021	0.7902±0.0059	**0.8768±0.0090**
	Dataset 3	0.7142±0.0005	0.7349±0.0183	0.7182±0.0138	**0.7631±0.0095**	0.6985±0.0103
	Dataset 4	0.7012±0.0065	0.6289±0.0277	0.7498±0.0144	0.7948±0.0070	**0.8211±0.0200**
	Dataset 5	0.7971±0.0031	0.7425±0.0047	0.7761±0.0016	0.8248±0.0011	**0.8753±0.0052**
	Ave.	0.7866	0.7518	0.7872	0.7942	**0.8238**
Recall	Dataset 1	0.6565±0.0083	0.8308±0.0154	**0.9652±0.0080**	0.9379±0.0283	0.9280±0.0126
	Dataset 2	0.6603±0.0068	0.8451±0.0242	**0.9504±0.0012**	0.6910±0.0092	0.9041±0.0100
	Dataset 3	0.6313±0.0075	0.6951±0.0336	0.7612±0.0237	0.6745±0.0065	**0.8238±0.0425**
	Dataset 4	0.6445±0.0046	0.5863±0.0638	0.6988±0.0143	0.7007±0.0052	**0.7945±0.0249**
	Dataset 5	0.7194±0.0014	0.8691±0.0035	0.8659±0.0030	0.7304±0.0006	**0.8714±0.0050**
	Ave.	0.6624	0.7652	0.8483	0.7469	**0.8644**
Accuracy	Dataset 1	0.7604±0.0027	0.8319±0.0170	**0.8933±0.0020**	0.8488±0.0136	0.8736±0.0112
	Dataset 2	0.7687±0.0032	0.8264±0.0107	**0.8976±0.0018**	0.6965±0.0057	0.8885±0.0069
	Dataset 3	0.6635±0.0038	0.7194±0.0061	0.7302±0.0044	0.6745±0.0065	**0.7338±0.0116**
	Dataset 4	0.6542±0.0044	0.6095±0.0138	0.7322±0.0092	0.7007±0.0052	**0.8104±0.0158**
	Dataset 5	0.7428±0.0030	0.7837±0.0030	0.8081±0.0010	0.7304±0.0006	**0.8736±0.0033**
	Ave.	0.7179	0.7542	0.8123	0.7302	**0.8360**
F1-score	Dataset 1	0.7421±0.0048	0.8315±0.0082	**0.9005±0.0020**	0.8614±0.0077	0.8802±0.0102
	Dataset 2	0.7495±0.0051	0.8295±0.0094	**0.9044±0.0016**	0.6565±0.0071	0.8970±0.0068
	Dataset 3	0.6702±0.0019	0.7110±0.0095	0.7379±0.0043	0.6359±0.0072	**0.7553±0.0164**
	Dataset 4	0.6716±0.0054	0.5881±0.0264	0.7226±0.0091	0.6636±0.0057	**0.8073±0.0165**
	Dataset 5	0.7563±0.0022	0.8007±0.0020	0.8186±0.0011	0.6923±0.0007	**0.8733±0.0032**
	Ave.	0.7179	0.7521	0.8168	0.7019	**0.8420**
AUC	Dataset 1	0.9247±0.0012	0.8846±0.0060	0.9292±0.0016	0.9293±0.0120	**0.9297±0.0112**
	Dataset 2	0.9352±0.0011	0.8918±0.0055	0.9389±0.0015	0.8893±0.0136	**0.9474±0.0069**
	Dataset 3	0.7883±0.6735	0.7940±0.0049	0.8229±0.0025	**0.8493±0.0130**	0.8235±0.0116
	Dataset 4	0.7823±0.0069	0.6421±0.0122	0.8047±0.0095	**0.9024±0.0105**	0.8866±0.0158
	Dataset 5	0.8826±0.0031	0.8156±0.0020	0.8903±0.0010	**0.9609±0.0013**	0.9458±0.0033
	Ave.	0.8626	0.8056	0.8772	0.9062	**0.9066**
AUPR	Dataset 1	0.8852±0.0006	0.8904±0.0084	0.9208±0.0028	**0.9290±0.0155**	0.9001±0.0153
	Dataset 2	0.9013±0.0035	0.8926±0.0049	0.9049±0.0028	0.8956±0.0128	**0.9262±0.0127**
	Dataset 3	0.7520±0.0006	0.7936±0.0062	0.8081±0.0038	**0.8560±0.0162**	0.8005±0.0112
	Dataset 4	0.7585±0.0119	0.6629±0.0190	0.8032±0.0104	0.6683±0.0061	**0.8767±0.0226**
	Dataset 5	0.8698±0.0032	0.7943±0.0019	0.8731±0.0016	**0.9596±0.0021**	0.9374±0.0043
	Ave.	0.8334	0.8067	0.8620	0.8617	**0.8882**
